# Epidemiology of Oral Cavity Cancers in a Country Located in the Esophageal Cancer Belt: A Case Control Study

**Published:** 2012

**Authors:** Babak Saedi, Ebrahim Razmpa, Masoomeh Ghalandarabadi, Hamidreza Ghadimi, Farnaz Saghafi, Mahshid Naseri

**Affiliations:** 1 *Department of otorhinolaryngology, Tehran University of Medical Sciences, Tehran, Iran*; 2 *General Practitioner and Medical Researcher, Health Researchers R&D Institute, Tehran, Iran*; 3 *Medical Researcher, Faculty of Medicine, Tehran University of Medical Sciences, Tehran, Iran*; 4 *Dentist, Oral Medicine Research Center, Tehran University of Medical Sciences*; 5 *Medical Student, Tehran University of Medical Sciences, Tehran, Iran*

**Keywords:** Cancer, Esophageal cancer, Epidemiology, Iran, Oral cavity

## Abstract

**Introduction:**

As one of the most common cancers among head and neck malignancies, cancer of the oral cavity probably has some variations in countries with a high prevalence of esophageal cancer.

**Materials and Methods:**

Patients with oral cavity cancer who were treated at two tertiary referral centers from January 1999 to January 2009 were included in this study. In addition to demographic data, information regarding personal and family history of head and neck cancer, use of dentures, presence of immune deficiency, consumption of alcohol, and incidence of cigarette smoking was collected. Additionally, a history of opium usage was obtained from the participants in this study. Moreover, an appropriately matched control group was selected for comparisons between the risk factors.

**Results:**

A total of 557 patients were entered into this study over a 10-year period, of whom 219 (39.3%) were female and the remaining 338 (60.7%) were male. The tongue was the most common site of cancer and 9% of the patients had a history of opium abuse, but more than half of the patients did not have any recognized risk factors. The incidence and stage of cancer had a significant relationship with cigarette smoking (P= 0.013).

**Conclusion:**

Tongue cancer in non-smokers is the predominant pattern of oral cavity cancer in Iran.

## Introduction

Cancer of the oral cavity is one of the most common cancers among head and neck malignancies (1, 2). Moreover, its particular location may enable physicians to detect it early enough to prevent or lower its morbidity. On the other hand, recognizing and eliminating potential risk factors is another strategy for preventing the occurrence of cancer. Over the years, many researchers have tried to find the most important risk factors, of which smoking and alcohol consumption are the most notorious culprits (1-9). However, genetic factors and tobacco chewing also play an essential role (1, 2, 4, 6, 7, 10, 11).

Despite the prevalence of oral cavity cancer in the world, its importance is not equal in different countries. Therefore, there is a huge discrepancy in morbidity in different geographical locations. The Iranian population has one of the highest rates of esophageal squamous cell carcinoma in the world (12). The cause of these extraordinary rates has been investigated (13) and it has been noted that the main risk factors include a poor diet, genetic susceptibility, and opium consumption (13). Moreover, the low rate of alcohol consumption in this area causes different patterns of other cancers (14). The above-mentioned risk factors are also common among upper gastrointestinal (GI) cancers. Considering the high rate of esophageal cancer in Iran and its possible correlation with other GI cancers, this study was designed to investigate the epidemiological pattern of oral cavity cancer in Iran.

## Materials and Methods

*Study subjects:* Patients with cancer of the oral cavity (a squamous cell carcinoma) who were treated at two tertiary referral centers (Imam Khomeini and Amir Alam Hospital) from January 1999 to January 2009 were included in this study. Data were taken from medical reports of patients with confirmed diagnoses who were registered during the study period. There was no randomization in this study, and all eligible patients were enrolled during the study period. Patients with oropharyngeal cancer were excluded from this series as were patients with other head and neck cancers, due to the possible higher prevalence of risk factors among those patients. In addition, 300 normal age- and sex-matched subjects with the same socioeconomic status were selected from normal individuals during the same period. 

*Ethical approval:* The protocol of this study was approved by the Institutional Review Board of the Tehran University of Medical Sciences. Detailed information about the study was given to the participants and written informed consent was obtained from each one. All aspects of the study were conducted according to the Declaration of Helsinki.

*Study variables: *In addition to demographic data, information regarding personal and family history of head and neck cancer, use of dentures, and presence of immune deficiency were collected. Furthermore, individuals were divided according to their self-reported consumption of alcohol (ever/never), smoking of cigarettes, and smoking of opium. Additionally, a history of opium usage was taken from each participant in this study. Opium dependency was defined according to the "Diagnostic and Statistical Manual of Mental Disorders, Fourth Edition Revised, criteria for opium dependency and opium consumption" as someone who has been opium dependent for at least 5 years. Tumors were categorized based on their locations. Data collection was conducted with the same methods under the supervision of the senior author.

*Statistical analysis: *Data were analyzed using SPSS 11.5 for Windows (SPSS Inc., Chicago, IL, USA). The chi-squared test was used to evaluate ratios and Student’s *t*-test to compare the average values.

 Fisher’s exact test and ANOVA were also used. Values were evaluated using descriptive statistical methods (mean ± standard deviation [SD]) and results were significant if P< 0.05.

## Results

A total of 557 patients were included in this study, of whom 219 (39.3%) were female and the remaining 338 (60.7%) were male. The mean age of the participants was 60.8 ± 14.7 years old, ranging from 25 to 95 years. Of all participants, 332 (59.6%) were illiterate or had a primary school education while the rest had higher levels of education. A previous history of head and neck cancer was present in 9 (1.62%) patients, consisting of cancer of the oral cavity in 5 (0.9%) patients and other head and neck cancers in the rest. Moreover, 15 (2.7%) patients had a positive family history for other cancers. A matched control group was selected for comparison. The characteristics of the two groups are summarized in (Table 1).

**Table 1:** Characteristics of the patients and controls subjects included in the study

	Patients	Control Subjects	*P* value
Age	60.8 ± 14.7 years	60.4 ± 16.3 years	0.99 (*t*-test)
			
Sex (female/male)	219/338 (39.3%/60.7%)	111/189 37.5%/62.5%)	0.87 (χ^2^)
			
Family history of head & neck cancer	9 (1.6%)	6 (2.0%)	0.5 (Fisher’s exact)
			
Alcohol consumption	18 (3.8%)	7 (2.3%)	0.63 (χ^2^)
			
Immune deficiency	5 (0.9%)	3 (1.0%)	0.33 (χ^2^)
			

**Fig 1:**
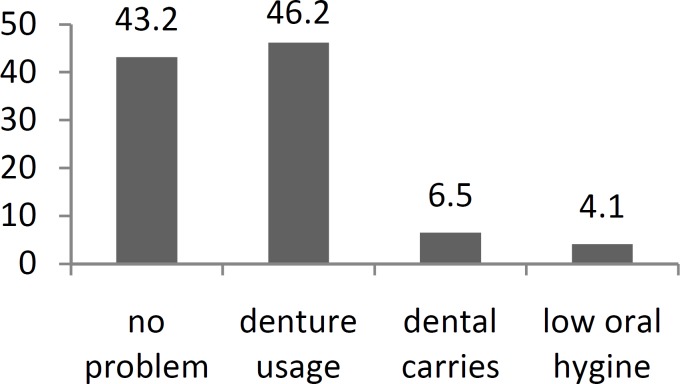
The distribution of dental problems in the evaluated patients

A total of 18 (3.2%) patients reported consuming alcoholic drinks; however, all had consumed alcohol one to three times a week for less than 20 years. Also, 52 (9.3%) had a history of opium addiction. Those who were current cigarette smokers smoked an average of 15 ± 7 cigarettes per day and had done so for an average of 30 ± 12 years. Dental problems were also evaluated as a potential risk factor for oral cavity cancer and the results are shown in (Figure 1). The most common dental problem was the use of dentures, which occurred in 46.2% of patients.

**Fig2:**
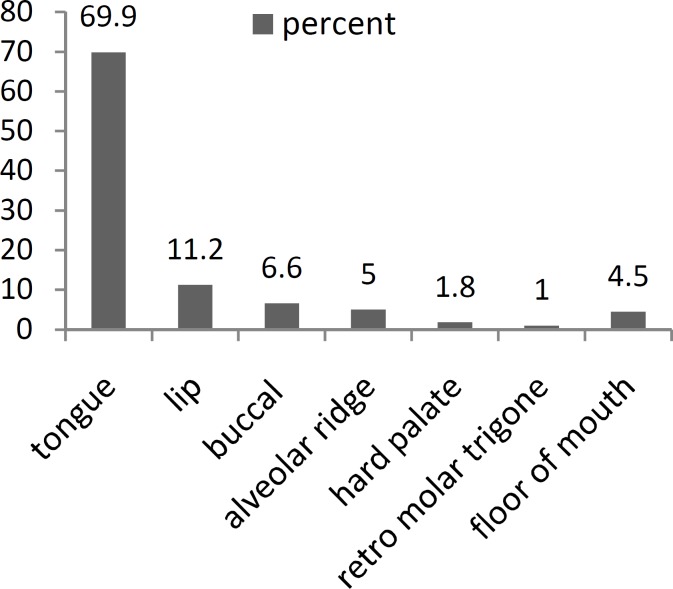
The distribution of different types of oral cavity cancer

**Fig 3:**
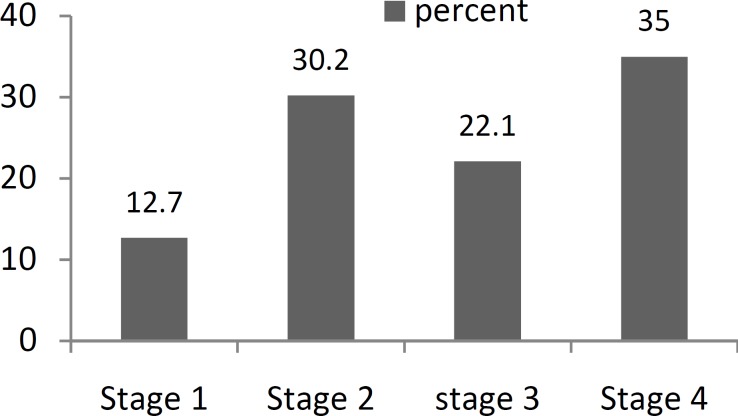
Distribution of the four stages of oral cavity cancer

The distribution of oral cavity cancer is summarized in (Figure 2). The most common site for oral cancer was the tongue, which occurred in 69.9% of patients. There were also 5 (0.9%) cases of gum cancer among the evaluated patients. The mean duration between the first presentation of cancer and diagnosis was 10 ± 8 months. There was no significant elationship between age (ANOVA, P=0.84), opium addiction (χ^2^, P=0.13), oral disease (χ^2^, P=0.27), and smoking (χ^2^, P=0.55) with the tumor location. Additionally, the status of the tumors was categorized using the TNM staging method according to a physical examination and pre-treatment images. The results of this evaluation are shown in (Figure 3). The relationship between tumor staging and the different sites was significant (Fisher’s exact test, P=0.013). A higher rate of advanced stage tumors was noted in the floor of the mouth, retro molar trigon, and buccal mucosa compared with the other sites of oral cancer. 

Although we failed to demonstrate a significant relationship between sex (χ^2^), age (ANOVA), and tumor staging, there was an almost significant relationship between staging and cigarette smoking (P=0.062). 

Logistic regression analysis of the variables (Table 2) indicated that there was a significant association between cigarette smoking and the occurrence of cancer of the oral cavity.

**Table 2:** The relationship between various risk factors and the development of oral cavity cancer

	**Standard Coefficient**	**T**	**P **
Age	-0.003	0.039	0.969
Gender	0.061	0.673	0.502
Alcohol	-0.014	0.162	0.871
Smoking	-0.061	0.662	0.009^*^

## Discussion

As a common head and neck cancer, oral cavity cancer has a hugely variable frequency in different parts of the world (1). The average incidence of oral cavity cancer is half a million new cases around the world, which can present an important health problem in some geographical locations (1). Compared to the rest of the world, Iran has some special distinctions. First, it is located in the region with the highest rate of esophageal cancer (13, 14). Second, the rate of alcohol consumption in Iran is low because of various social factors, mainly religious beliefs. Finally, there is a high rate of opium addiction among oral cavity cancer patients (15). To our knowledge, the epidemiology of oral cavity cancer in Iran has not yet been thoroughly studied. The present study was conducted at Imam Khomeini and Amir Alam Hospitals, the two largest university-affiliated referral centers, and the locations where most cases of oral cavity cancer are treated. This study of 557 new cases from these centers over a 10-year period is acceptably representative of the epidemiologic pattern of oral cavity cancer in Iran.

Among the possible oral cavity cancer sites, the tongue was the most common site with 70% of primary cancers occurring in this location, which is a similar rate to that observed in European populations (1). Lip cancer was the second most common cancer in our series, a location that is the most commonly affected site only in some parts of the world such as Canada and Australia (1). More than 60% of the patients were male, which is consistent with most series. Similarly, the mean age of the patients was 60, which is also well-matched with other series (1,6,7,15). For the two most common oral cavity cancer risk factors, only 35% of the patients had a history of tobacco consumption and a mere 3.2% drank alcohol. However, an outstanding feature of this series was the high rate of opium abuse (9%) in the patients included in the study, which has been proposed as a possible cause of several cancers such as esophageal and laryngeal cancer (16). Therefore, the possible role of opium addiction can be considered in this cancer.

A total of 57% of the cancers detected were advanced stage (T3 and T4) cancers, indicating a tendency towards late diagnosis and the aggressive nature of the tumors in this series (6). Furthermore, the use of dentures and smoking had a significant association with oral cavity cancer and tobacco use was also associated with more aggressive tumors. These findings confirm the role of tobacco consumption as an etiologic factor for oral cavity cancer (1,2,7,17) and also suggest the role of dental disease in inducing this type of cancer (8). However, the role of the above mentioned risk factors in causing cancer is not certain since more than half of the cases did not have any recognized predisposing factors. Therefore, these cases should be categorized as oral cavity cancers in non-smokers and further possible etiologic factors considered. However, the rate of oral cavity cancers in non-smokers was conspicuously higher is this study than in other series (15,18).

Considering the high rate of other cancers, such as esophageal cancer, in Iran and the possible role of common etiological factors in the development of head and neck cancers, these factors can also be proposed as risk factors for oral cavity cancer (18). As predisposing factors for esophageal cancer, genetic factors, nutritional deficiencies (3,19,20), and opium addiction may play a role in the development of oral cavity cancer. Due to variations in head and neck cancers in Iran and their notable difference with other parts of the world (14), it is suggested that more studies be conducted on cancer epidemiology in Iran.

One of the shortcomings of this study is the location of the study, which was performed in two academic referral hospitals and could have produced possible differences compared with the natural epidemiology of cancer in Iran. Because of the fact that the control subjects were selected from among normal subjects, who were referred to these centers, the mentioned problem may have been diminished. Moreover, Iran is a very vast country with a huge population, so despite the importance of the location of the study the characteristics of the study group may not be exactly the same as the cancer characteristics all over the country. For example, many patients in the east part of Iran go to Mashad University centers and patients from the southern parts of Iran choose to go to Shiraz University centers to be treated. However, as we don’t have a complete cancer registry Iran, the final results of the current study are of use. 

## Conclusion

Tongue cancer in non-smokers is the predominant pattern of oral cavity cancer in Iran. Similar to other studies, smoking had an etiologic role in oral cavity cancers in this series. In future studies, a more thorough patient history should be performed to reveal other possible risk factors.
